# From Antibody Repertoires to Cell-Cell Interactions to Molecular Networks: Bridging Scales in the Germinal Center

**DOI:** 10.3389/fimmu.2022.898078

**Published:** 2022-05-04

**Authors:** Haripriya Vaidehi Narayanan, Alexander Hoffmann

**Affiliations:** Signaling Systems Laboratory, Department of Microbiology, Immunology, and Molecular Genetics, and Institute for Quantitative and Computational Biosciences, University of California, Los Angeles, Los Angeles, CA, United States

**Keywords:** germinal center, B-cells, multi-scale dynamics, mathematical modelling, affinity maturation, antibody repertoire, rational vaccine design, precision vaccination

## Abstract

Antibody-mediated adaptive immunity must provide effective long-term protection with minimal adverse effects, against rapidly mutating pathogens, in a human population with diverse ages, genetics, and immune histories. In order to grasp and leverage the complexities of the antibody response, we advocate for a mechanistic understanding of the multiscale germinal center (GC) reaction – the process by which precursor B-cells evolve high-affinity antigen-specific antibodies, forming an effector repertoire of plasma and memory cells for decades-long protection. The regulatory dynamics of B-cells within the GC are complex, and unfold across multiple interacting spatial and temporal scales. At the organism scale, over weeks to years, the antibody sequence repertoire formed by various B-cell clonal lineages modulates antibody quantity and quality over time. At the tissue and cellular scale, over hours to weeks, B-cells undergo selection *via* spatially distributed interactions with local stroma, antigen, and helper T-cells. At the molecular scale, over seconds to days, intracellular signaling, transcriptional, and epigenetic networks modulate B-cell fates and shape their clonal lineages. We summarize our current understanding within each of these scales, and identify missing links in connecting them. We suggest that quantitative multi-scale mathematical models of B-cell and GC reaction dynamics provide predictive frameworks that can apply basic immunological knowledge to practical challenges such as rational vaccine design.

## Introduction

Effective immune responses to pathogens are characterized by antibody production in the short term, and generate immune memory against that specific antigen in the long term. Antibodies are an important mediator of the immune response to infection, with the ability to bind various pathogens and neutralize their infectivity or accelerate their clearance. Eliciting repeated, specific, and potent antibody responses to a given pathogen is also a primary goal of vaccination, and the desired outcome of most vaccine development strategies.

There are two main challenges in mounting the appropriate antibody responses to infection or vaccination, arising from the variations intrinsic to both pathogen and human populations. First, pathogens with high mutation rates result in multiple and fast-evolving strains, which lead to immune evasion or antigenic drift, hampering responses to chronic infections or development of immunization strategies. This is reflected in the difficulty of developing effective vaccines for HIV, malaria, or dengue, despite the urgent need to address these high-mortality public health challenges. Here, we need vaccine designs that provide optimal coverage across pathogenic strains – for example, through broadly neutralizing antibodies, or by producing a diverse repertoire, or by minimizing deleterious cross-reactive effects. Second, human variations in genetic composition, age, gender, antigen and environmental exposure history, tonic inflammatory setpoint, and myriad other physiological parameters drastically vary our immune responses, even to the same pathogen. This means that antibody responses and vaccine efficacy are both highly variable across the human population, and extremely challenging to predict. To maximize protective coverage, we further require precision vaccination, through personalized cocktails of co-stimulatory or other pharmacological compounds that act on the molecular scale. Addressing this dual challenge of pathogenic and human variation prompts us to ask whether antibody response prediction and the rational development of vaccination strategies are possible – specifically, by leveraging a fundamental understanding of cell biological and immunological processes.

**Figure 1:**
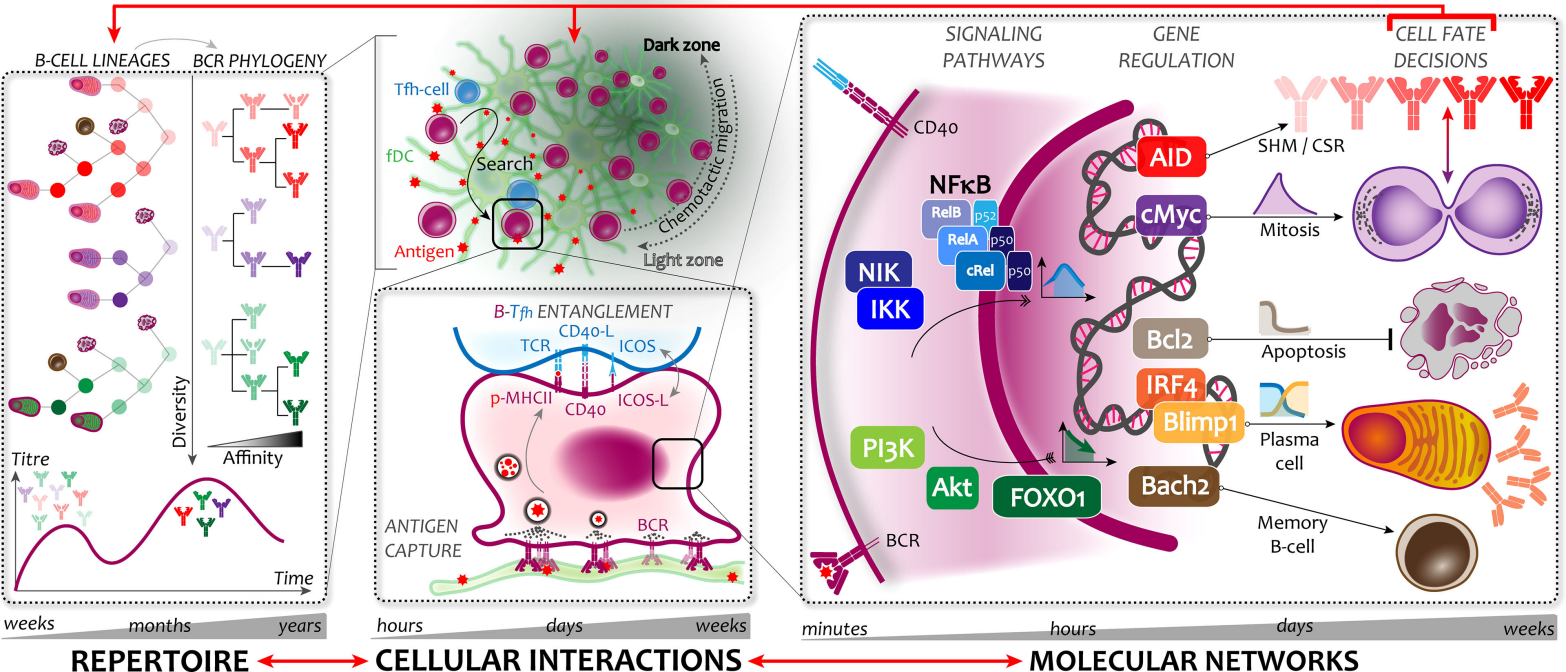
Germinal center dynamics at (Left) repertoire, (Center) cellular interaction, and (Right) molecular network scales. Red arrows indicate connections between scales that remain to be fully explored. *(L) Repertoire scale:* Affinity maturation is shaped by B-cell lineages arising from various founders (shown in different colors) that contribute to clonal diversity of the response, producing plasma and memory cells. Each lineage corresponds to a BCR sequence phylogeny, whose evolution results in increased affinity over time (shown in darker shades). The net outcome is the phased production of antibodies over primary and subsequent responses, with lower titers of more diverse but lower affinity antibodies at early times, followed by a high-titer, more focused, high-affinity repertoire at later stages. *(C) Cellular interaction scale:* (Top) The germinal center has a spatially segregated structure, with B-cells migrating between zones. (Bottom) The light zone is the site of competitive selection, through affinity-based antigen capture and endocytosis, followed by antigen presentation to T-follicular helper cells and B-Tfh entanglement. Recognition of peptide-MHCII complexes by TCRs on Tfh-cells leads to increased contact and attachment, reinforced by ICOS-ICOSL positive feedback, and leading to CD40 signaling in B-cells. B-cells selected *via* sufficient CD40 signaling return to the dark zone to execute various cell fates. *(R) Molecular network scale:* Signaling in B-cells is mediated by multiple pathways, whose temporal dynamics effect their different cell fate outcomes. Among these, nuclear exit of FOXO1 is implicated in licensing the LZ phenotype, enabling antigen and Tfh encounters. Nuclear translocation of NFκB induces transcription factors that regulate B-cell survival, division, and differentiation. B-cell division is accompanied by hypermutation of BCR sequences, which influence future antigen and Tfh encounters, hence the subsequent B-cell fates that shape their lineages, thus ultimately driving the antibody and memory repertoire evolution.

Antibody responses arise from the evolution of precursor B-cells into their effector and memory counterparts. This occurs through a highly dynamic process, whose basic underlying science can be understood at three distinct scales. **Figure 1** provides a graphical overview representing these different scales. First, at the level of an individual organism, spanning timescales of weeks to years, are the temporal dynamics of the antibody repertoire itself. This includes the phasing of low versus high affinity antibody over time in a process known as affinity maturation, and the development of memory and long-lived plasma cells. The second dynamical scale of multi-cellular interactions within lymph nodes occurs over timescales of days to weeks. Here, the repertoire is generated within specialized transient structures called germinal centers (GCs), where interactions between antigen, B-cells, and helper T-cells are intricately coordinated to enable the evolution of antigen-specific antibodies. The third dynamical scale, at the level of the molecular network within each B-cell, enables the B-cell fate decisions subsequent to these interactions, on short timescales of minutes to days. These network dynamics involve both signaling from receptors as well as genetic and epigenetic regulation, to direct and execute various B-cell fates. The topology and temporal evolution of this intracellular network, arising from the abundances of protein molecules and their biochemical reaction kinetics, determines the propensity and ability of each B-cell to survive, proliferate, or differentiate in response to antigen. Taken together, these three regulatory levels, operating at different spatial and temporal scales, collectively determine the overall immunological response to infection or vaccination.

Leveraging immunological knowledge of the antibody response for clinical applications begins with a mechanistic understanding of B-cell dynamics in the germinal center. This requires not only studies at each of the three individual dynamical scales, but also on how processes at one scale impact regulation at a different scale. Often, when studies are confined to a higher scale, regulatory control at lower scales is abstracted into heuristics or empirical rules, which can limit predictive or explanatory power. On the other hand, for a complete mechanistic view, the rules governing phenomena at a higher scale must emerge naturally from known regulatory mechanisms at lower scales. For example, the antibody repertoire may be accounted for in terms of the phylogeny of antibody sequences and targeted amplification of some B-cell lineages, which may be further explained by mutation, selection, cell death and division decisions, which are in turn driven by cellular interactions, signaling, and intracellular molecular networks. Developing such a coherent and comprehensive mechanistic framework for the antibody response necessitates knowing how its different dynamical scales are mutually interconnected.

In this review, we summarize the substantial scientific advances at each of the three scales of the antibody response – affinity maturation of the repertoire, germinal center interactions, and B-cell-specific molecular networks. We then make the case that although much is known at each scale, there are critical missing links between the different scales. A predictive mechanistic framework should connect these links to show how antibody response properties build up from the molecular to the organism level. We thus advocate for a quantitative multi-scale systems immunology approach to understanding germinal center B-cell dynamics, to incorporate basic science towards applications such as rational vaccine design.

## Dynamics at the Repertoire Scale: Affinity Maturation of B-Cells

The most striking phenomenon in the B-cell immune response to infection or vaccination is affinity maturation. Here, a random starting pool of low affinity antibodies gives rise over time to highly antigen-specific, high affinity antibodies, as first outlined by Burnet’s clonal selection theory (1). Briefly, naïve B-cells emerge from the bone marrow, each expressing on its surface exactly one out of trillions of possible B-cell antigen receptor combinations, recombined from a set of germline sequences during hematopoiesis. These are then exposed to the immunizing antigen, and a subset of B-cells with receptors that preferentially bind this antigen is selected to propagate the response. Subsequent rounds of mutations of these receptors serve to explore the space of binding affinities across variants within each B-cell lineage. Competition between these variants *via* interactions with antigen and T-cells selects for high affinity receptors. The selected B-cells are then clonally expanded by proliferation, along with further diversification, while the others do not survive. Some of the selected clones differentiate into both short- and long-lived plasma cells (SLPCs and LLPCs), which secrete their receptors as soluble antibody. Others may differentiate into memory B-cells (MBCs) which survive for decades. These MBCs together with the LLPCs form the B-cell memory compartment that initiates and effects secondary responses to future antigen exposures.

This response is associated with characteristic temporal dynamics (2). In its early stages within 3 to 5 days, low affinity neutralizing antibodies are produced, which provide rapid but less effective protection. This stage also generates the MBCs, which serve as a record for future encounters with the same pathogen. The later stages of the response, after about 7 days, generate high affinity antibodies. These are secreted by LLPCs – the complementary form of immune memory – which also survive in the bone marrow for many years providing continuous protection. Beyond the primary response generated upon the initial exposure, secondary responses can be generated by a subsequent infection, or a booster dose of vaccine. The secondary response times are shortened due to starting with memory instead of naïve B-cells. These generate even stronger protection, with both the quantity and affinity of antibody production being higher than the primary response, thus building upon it.

We can define several relevant dynamical features of antibody repertoire evolution during the B-cell response to infection or vaccination, depicted in the left panel of **Figure 1**. The most immediately relevant is quantity – both of serum titers of secreted antibody, and the number of generated memory cells including MBCs and LLPCs. Ideally, a strong response produces not only sufficient titers of neutralizing antibody to eliminate the antigen during the short term, but also long-lived memory cells in enough numbers to protect against subsequent infections. Second, in addition to quantity, the quality of antibody produced also matters. This can be quantitively defined by the distribution of affinities of the various sequences comprising the repertoire. In general, this distribution is expected to become higher and narrower over time as the response matures. Additionally, the speed at which high-affinity antibodies appear also impact response dynamics and overall outcomes. Recent studies on mortality in SARS-CoV2 infection underscore this point, where both surviving and deceased patients were able to generate high affinity antibodies, but the timing of when these antibodies appeared in the serum was a critical determinant of patient survival (3). Going further, the specific antibody sequences that are generated are also of great interest – particularly in determining which clonal lineages give rise to known high affinity or broadly neutralizing antibody sequences, in order to design vaccine targets that preferentially elicit these sequences. Advances in large-scale repertoire sequencing as well as inference-based computational reconstruction of antibody phylogenies (4, 5), together with analysis techniques and metrics for detecting signatures of selection (6), have enabled high-resolution studies of how the sequence composition of the repertoire changes in response to infection or vaccination. Finally, ensuring sufficient diversity of the generated repertoire is also critical to ensure robust protection against future infections, as a safeguard against immune evasion and antigenic drift. A complementary aspect is the specificity of each antibody against its target, which influences both diversity and protective coverage, by determining the overlap between recognized targets and potential gaps in epitope recognition.

The dynamical scale of the organism-level antibody repertoire is the most immediate and relevant for vaccinology. Several active areas of research aim to shape or exploit these dynamical characteristics to improve vaccination strategies. Host-pathogen interactions are a major focus in the generation of broadly neutralizing antibodies (bnAbs) against rapidly mutating pathogens, such as the influenza virus or HIV, which bind common antigen epitopes shared by multiple strains (7). Quantitative modelling has revealed that one strategy to elicit these bnAbs is through sequential immunization with a variety of strains (8). Complementary models, which integrate antibody structure prediction and affinity estimates with GC processes, further suggest that antigen cocktails play an important role in inducing cross-reactivity across similar epitopes, with sequential cocktail immunizations yielding the highest affinity antibodies (9). Another important focus area is to improve the efficiency of affinity maturation. Experimental evidence has corroborated mathematical modelling predictions that continuous availability of antigen throughout the response, particularly in exponentially increasing doses mimicking natural infection, promotes better affinity maturation during vaccination (10). A third topic of interest is the optimization of adjuvants, which are vaccine additives generating the inflammatory conditions that promote an effective B-cell immune response, but which may also boost the innate immune responses intertwined with antibody generation (11). Finally, it is also relevant to consider the effect of other organism-level dynamical processes on the antibody response – for example circadian rhythms, where research shows increased generation of plasma cells when antigen is administered earlier in the day (12, 13).

In the examples described above, quantitative mathematical models of affinity maturation have assumed critical importance in the rational development of vaccination strategies. These models employ heuristics based on empirical observations to capture the dynamics of affinity maturation and guide global antigen-specific parameters at the organism level, such as the temporal dosing and composition of immunizing antigen. However, it remains a challenge to relate these models and strategies to other host-specific factors, such as genetic or epigenetic make-up, particularly in a predictive manner. Pioneering approaches in systems vaccinology have been developed to relate baseline physiological parameters in individuals to their post-vaccination outcomes (14). The next step is to capture the actual dynamical variation of these parameters over time during the antibody response. This requires a synthesis of repertoire-scale affinity maturation dynamics with its mechanistic details at the cellular and molecular scales – respectively, the interactions of B-cells within lymphoid tissues, and their internal signaling and gene regulatory networks.

## Dynamics at the Cellular Scale: Coordinated Interactions in Germinal Centers

The process of affinity maturation is built upon the various cellular interactions that enable competitive selection between B-cells. These take place within germinal centers (GCs) – specialized structures within lymph nodes that spontaneously arise during infection or vaccination responses. The GC serves to organize B-cell interactions with antigen and other cells into a dynamical process distributed over both space and time. However, a major challenge in observing, measuring, or perturbing these interactions is the difficulty of reproducing GC structures and functions outside living organisms. Hence, the key contributions to our understanding mainly come from intravital multi-photon imaging studies capable of deep tissue optical penetration, which directly observed and tracked individual B-cells in their native environments within lymph node follicles (15, 16). Here, we briefly summarize this knowledge through a particular focus on B-cell-specific interactions with the other participants in affinity maturation, which are of primary relevance to vaccine design and development. The middle panel of **Figure 1** provides a graphical representation of the dynamics described in this section.

### Interaction With Stromal Cells: Spatial Segregation and Inter-Zonal Migration

A striking characteristic of the GC is its spatial segregation into two regions, known from older histological studies as the light zone and dark zone due to their differing densities of cells (17). The light zone is the region of antigen display and competitive selection *via* B-cell interactions. The dark zone is the site of B-cell proliferation, and also conjectured to be where other fate choices such as differentiation into antibody-secreting cells (ASCs, including plasmablasts, SLPCs, and LLPCs) are made. Structurally, these two zones arise from a foundation of different stromal cells that have recently been characterized using single-cell RNA-seq methods (18). The reticulate cells in the dark zone are primarily defined by their expression of the chemokine CXCL12, forming a network of variable morphology (19), with a poorly understood functional role outside of localizing proliferative B-cells.

By contrast, the stroma of the light zone is well-characterized. These are called follicular dendritic cells (FDCs), and form a branched interconnected network that serves the important function of antigen presentation to B-cells (20). FDCs express increased levels of the B-cell survival factor BAFF (21), and have CR1/CR2 (22) and Fcγ receptors (23) on their surface, with which they respectively bind complement- and antibody-opsonized antigen into immune complexes (ICs) (24). B-cells walk along the processes of this FDC network, picking up antigen with their B-cell receptors for affinity discrimination and competitive selection (25). Antigen is retained on the surface of FDCs for several weeks at a time, depending on the size of the antigen particles. This allows FDCs to serve as a critical reservoir driving the GC reaction, since GC efficiency is sharply impacted by the duration of antigen availability (26). The stromal network is adversely impacted by aging, diminishing its capacity to support the GC reaction, which is thought to underly poorer responses in older adults (27).

Migration between zones is driven by gradients of two different chemokines, CXCL13 and CXCL12, respectively expressed by the light and dark zone stroma (28). B-cells upregulate different chemokine receptors at different points of the GC cycle, allowing them to transition between the two zones and execute different transcriptional programs in each. The kinetics of migration across and within these zones have been quantified with intravital imaging studies, including those where cells in a specific region were labelled with photoactivatable GFP for subsequent tracking (29). This crossing over between zones is termed cyclic re-entry, and has been captured by detailed mathematical models (30). Measurements showed that an initial encounter with a Tfh-cell led to about 12 hours of residence time in the light zone, which is then followed by movement to the dark zone and proliferation over several days. Conversely, the transition from the dark to light zone appears not to be induced by any signals, but rather driven by a B-cell-intrinsic “timer” program (31). Intravital photoactivation has also quantified the rate of this timed migration, with about 50% of dark zone cells transitioning to the light zone over 4 hours (29). The spatial segregation between GC zones creates a time delay between the B-cell interactions driving selection and their subsequent fate decisions. Theoretical studies have demonstrated that this temporal separation is an important requirement for driving efficient and optimal affinity maturation (32).

### Interaction With Antigens: Affinity Discrimination and Surface Display

Interactions between B-cells and antigen occur *via* the B-cell receptor (BCR), which recognizes intact antigen displayed on FDCs in the light zone. B-cells execute a random walk in this region, with their BCRs binding to the antigen and pulling on it. Biomechanical studies have shown that GC B-cells have significant responsiveness to membrane-immobilized antigen (33). Tensile forces applied at the BCR allow the B-cell to rearrange their cytoskeleton in order to exert pulling forces proportional to their affinity (34). This enables affinity discrimination, where high affinity cells are able to exert more force and take up more antigen. Antigen engagement in GC B-cells differs markedly from those of naïve and memory B-cells, primarily due to differences in their BCR-antigen synapse architecture (35, 36). While naïve B-cells simply engage antigen above a threshold affinity, the specialized synapse architecture in GC B-cells localizes at a few contact points, allowing a greater range of antigen pulling forces and hence better affinity discrimination (37). Antigen pulled off the FDCs is then endocytosed by the B-cells. Peptide antigens are processed *via* antigen presentation pathways and displayed as peptide-MHCII complexes on the B-cell plasma membrane, to enable recognition by T-cells.

### Interaction With T-Follicular Helper Cells: B-T Entanglement

B-cells in the light zone encounter a limiting number of cells from a CD4+ T-cell subset known as T-follicular helper (Tfh) cells. The B-cells compete with each other to receive selection signals from these Tfh-cells *via* their CD40 receptors. Experiments that directly coated B-cells with surface antigen (bypassing the BCR) showed that these Tfh-induced CD40 signals are the primary driver of B-cell selection in the GC (38). Recent work indicates that the interactions with Tfh-cells “refuel” B-cells, promoting their survival in the DZ and enhancing the likelihood of their subsequent return to the LZ (39). The duration of contact with Tfh-cells increases with the amount of antigen displayed on the B-cell surface, resulting in a greater number of divisions on average (40). Higher affinity B-cells are able to physically pull away Tfh-cells from lower affinity B-cells, as a mechanism of direct competition between them (25). Further, the interaction between the receptor-ligand pair of costimulatory molecules – ICOS on Tfh-cells and ICOS-L on B-cells – sets up a feed-forward loop that upregulates the expression of CD40L on Tfh-cells and CD40 on B-cells (41). This means that both B-cells and Tfh-cells with a higher degree of interaction, initiated by surface antigen display, are driven towards prolonged contact durations, with a much larger contact area. Such indirect competition amplifies selectivity by ensuring that the highest affinity B-cells receive far greater amounts of Tfh-cell signals than lower affinity cells. Thus, the interaction of Tfh-cells with B-cells in the GC provides an indirect assessment of BCR affinity, and serves as the critical signal determining their survival and fate outcomes in the GC.

## Dynamics at the Molecular Network Scale: Signaling and Fate Decisions Within B-Cells

During successful competitive selection, the behaviors and fate choices of B-cells in the GC are enabled and executed by their internal signaling and gene regulatory networks. Signal transduction within GC B-cells is the lynchpin that connects their interactions with antigen or Tfh-cells to their subsequent cell fate outcomes. The connection between signaling and B-cell fate decisions within the GC is not fully understood, but an active area of research with considerable potential to impact vaccinology. Our current knowledge about these signaling and regulatory proteins comes primarily from genetic perturbation studies. Time course studies of signaling and fate regulation using biochemical assays have measured signaling activities and gene expression, with flow and image cytometry assays providing single cell resolution. Recently, live single-cell microscopy on B-cells stimulated *in vitro* has revealed their cell fate decision dynamics (42, 43). Snapshot assays like single-cell RNA-seq (44, 45) or other multimodal sequencing and proteomics analyses (46) applied to *in vivo* samples have contributed to knowledge of gene expression at various stages of the GC cycle. Here, we briefly describe some of the signaling pathways and gene regulatory circuits relevant to B-cell affinity maturation, organized by their functional roles and impacts relevant to vaccination, upon both the B-cell response and the overall antibody repertoire. The right panel of **Figure 1** gives a schematic representation of the dynamics at this scale.

### Signaling in the GC Context: The B-Cell Receptor and CD40

The first signal encountered by B-cells in the GC is *via* the BCR, upon binding to displayed antigen. The BCR doubles as both a signaling and an endocytic receptor, with its endocytic role discussed earlier in the context of antigen display. *In vitro*, ligation of the BCR in naïve B-cells produces transient signaling *via* multiple pathways including NFκB and PI3K/Akt which are pro-proliferative, as well as a pro-apoptotic signaling effect, which is also observed in the GC (47). Further, a single pulse of BCR stimulation has been shown to potentiate the pro-proliferative signaling in response to subsequent CD40 stimulation (48). In GC B-cells, signaling downstream of the BCR is attenuated compared to naïve B-cells, possibly due to increased phosphatase activity (49). Notably, in addition to PI3K attenuation, nuclear translocation of NFκB is absent even with strong BCR ligation in GC B-cells (36), indicating a “re-wiring” of their signaling network compared to naïve B-cells (50). The observed signaling activity comes through a short pulse of Syk phosphorylation following BCR ligation, leading to the rapid nuclear displacement of the transcription factor FOXO1 (50). As FOXO1 primarily maintains the dark zone phenotype (51), its nuclear inactivation facilitates the retention of GC B-cells within the light zone, for further encounters with Tfh-cells.

The second signaling interaction in the GC is when the CD40 receptor on B-cells is engaged by its corresponding ligand (CD40L) expressed on the surface of cognate Tfh-cells. While PI3K signaling downstream of CD40 remains attenuated in GC B-cells (50), CD40 engagement induces NFκB activity, with its nuclear translocation (36) and associated gene expression observed in a subset of light zone B-cells (29). The NFκB signals downstream of CD40 are transduced *via* both canonical and non-canonical pathways. The interaction between canonical NFκB signaling downstream of the BCR and non-canonical signaling has been previously understood in the case of the B-cell survival factor BAFF (52), and has yet to be explored for CD40. A detailed understanding of CD40 signaling is important because the nuclear activity of NFκB induces the transcription of anti-apoptotic factors, as well as other transcription factors driving proliferation and plasmablast differentiation, thus shaping both positive selection and the final effector repertoire.

### Determining Population Sizes Within the GC: Apoptosis vs Proliferation

Competitive selection in B-cells is driven by the two opposing processes of apoptosis and proliferation (53), directed by signaling within the light zone. These determine both the overall B-cell population size as well as the specific clonal lineages that are expanded to make up the emerging repertoire. Death and division in B-cells must be delicately balanced, in order to provide sufficient immune protection while avoiding adverse effects such as autoreactivity. Selected B-cells in the GC all appear to have roughly similar rates of proliferation, whereas their rates of apoptosis are highly affinity dependent (54). This indicates that selection within the repertoire may be more strongly driven by pruning the low affinity lineages *via* apoptosis, as opposed to expanding the high affinity lineages *via* proliferation.

Apoptosis is highly prevalent in the germinal center, with about half of all GC B-cells dying every 6 hours, as estimated using an *in vivo* reporter (55). Apoptotic B-cells are rapidly phagocytosed by tingible body macrophages to clear the GC of debris. The default outcome for light zone cells that do not receive Tfh-cell signaling is apoptosis, which is consistent with the *in vitro* observations of B-cell death following BCR ligation discussed above. Positively selected B-cells in the light zone receive anti-apoptotic signals, thought to be from NFκB activity driving the expression of Bcl-family transcription factors (56). In the dark zone, apoptosis appears to be dependent on somatic hypermutation processes, primarily serving to eliminate B-cells that fail to generate structurally stable or functional BCRs (55).

Positively selected B-cells in the GC undergo between one to six divisions, with larger numbers of divisions termed “clonal bursting”. Growth and proliferation in naïve and GC B-cells are both proportionally driven by the activity of the transcription factor cMyc (53, 57, 58), which is induced by NFκB (59, 60) and inhibited by FOXO1 (61). Thus, the clearance of nuclear FOXO1 upon BCR stimulation, coupled with the nuclear translocation of NFκB following CD40 stimulation, synergistically allows the upregulation of cMyc in positively selected B-cells (50). Selection signaling by Tfh-cells leads to cell growth *via* the mTORC1 pathway (62), while cell cycle entry is correlated to cMyc expression (57) and subsequent cyclinD3 induction (45). Clonal bursting is associated with stronger Tfh-signals, followed by greater levels of cMyc induction, a shorter S-phase of the cell cycle, and increased number of cell divisions (63). The relationship between spatial interactions in the GC and regulation of mTOR, cMyc, and FOXO1 has been explored using mathematical modeling, and suggests that separate signals are required to determine the two distinct outcomes of cell survival and the number of divisions (64). Paradoxically, although positive selection is associated with NFκB and cMyc, their expression is only observed in a limited subset of light zone B-cells (63, 65). Another interesting feature of the relationship between selection signaling and B-cell fate decisions is the spatial and temporal divide between them. Mostly (although not exclusively), selection occurs within the light zone, while proliferation takes place in the dark zone. Additionally, there is a time delay between contact with Tfh-cells and B-cell divisions, which indicates that B-cells can “remember” their interaction strengths to produce an appropriate number of divisions at later times.

### Diversifying the Repertoire: Somatic Hypermutation and Class Switch Recombination

B-cell proliferation in the dark zone is accompanied by somatic hypermutation (SHM) (66), where BCR sequences are altered, and the BCRs expressed on the surface are replaced by turnover (67). The BCR on a naïve B-cell consists of two chains – heavy and light – assembled from receptor-specific sequences called variable (V), diversity (D), and joining (J) sequences (note that light chains lack the D region). Within each naïve B-cell, the BCR is assembled by DNA recombination during development in the hematopoietic compartment, by randomly drawing one of each from a germline set of many individual V, D, and J sequences (68). This gives rise to myriad combinatorial possibilities that make up the naïve repertoire. In proliferating GC B-cells, the receptor sequences are targeted by the enzyme Activation-Induced Cytidine Deaminase (AID), which makes lesions in VDJ regions (69). These are then converted to point mutations by the error-prone DNA polymerase Polη (70). This mutation is estimated to occur at a rate of 1 per 1000 base pairs, corresponding roughly to one mutation per two B-cell divisions for the ~500 bp-long VDJ region (71, 72). This serves to diversify the repertoire in the GC and drive selection for increased affinity mutants.

AID is also implicated in a second process called Class Switch Recombination (CSR), where the constant region of the BCR is altered to a different isotype – most notably, from IgM to IgG in many high affinity GC B-cells and plasma cells (69). CSR plays an important role in controlling the dynamics of the antibody response, since the cytoplasmic tails of different constant region chains have differential immune signaling capabilities (73, 74). The relative timing of SHM and CSR is unclear, although it is known that lower levels of AID are required for CSR (75). Recent work has shown that CSR may actually take place prior to GC entry, with the GC serving as the primary site for SHM due to increasing levels of AID (76). Specific factors in the signaling and regulatory network are known to induce AID expression, such as the canonical subunits of NFκB (77), although the relationship between signals and CSR/SHM timing also remains fuzzy. Further investigation to pinpoint these relationships is important, since SHM and CSR shape the evolutionary dynamics of affinity across the B-cell effector repertoire.

### Differentiation Into Effector Cells: Antibody Secreting Cell and Memory B-Cell Generation

B-cells that survive in the GC not only proliferate and diversify their receptors, but also differentiate into antibody-secreting plasma cells and memory B-cells (MBCs), as the ultimate output of the GC reaction (2). Since these are the effectors of antibody immunity and long-lasting memory, producing sufficient numbers of long-lived plasma cells (LLPCs) and MBCs is the desired outcome of vaccination. The transition from a cycling GC B-cell to an antibody secreting cell (ASC) was explained with a gene regulatory circuit that functions as an irreversible bistable switch (78, 79). GC B-cells, which represent the first stable state of this circuit, are characterized by high expression of the transcription factors Pax-5 and Bcl-6. The second stable state, which corresponds to ASCs, is characterized by high levels of IRF4 and Blimp1. Since Bcl-6 and Blimp-1 mutually repress each other, these two states are therefore mutually exclusive. Further, since IRF4 and Blimp1 reinforce each other through a positive feedback loop, the transition to the ASC state cannot be downregulated, and hence becomes irreversible.

The signals within the GC that lead cycling B-cells towards ASC differentiation as opposed to proliferation are not yet clear, although Tfh-signaling strength is suggested to play a role (80). It is also unclear what directs a B-cell towards either ASC or MBC fate, and why this occurs in temporally distinct phases. ASCs tend to be of high affinity generally, although lower affinity ASCs may be generated in the early GC. Although some studies have shown that high affinity cells are directed to become ASCs (81), others indicate that these cells are instead preferentially expanded through division (82). There is also speculation that asymmetric distribution of antigen following B-cell division may favor ASC differentiation in the antigen-retaining progeny (83). Modeling studies combining regulatory networks with spatial cell-cell interactions have shown that a significant asymmetry in the distribution of antigen along with Blimp1 among daughter cells is consistent with the observed temporal switching between early memory and late plasma cell outputs (84). Further, the signals that direct differentiation into MBCs and their relationship to ASCs are also unclear. Studies suggest that precursor memory B-cells with low levels of the transcription factor Bach2 are predisposed towards ASC formation (85). Conversely, a low strength of selection signaling by Tfh-cells has also been implicated as the driver of Bach2 expression and hence MBC differentiation (86). Precursor memory B-cells that localize in the LZ have been identified and their gene expression profiles characterized, showing reduced levels of Bcl6 and cell cycle shutdown, coupled with increased expression of Bcl2 which mediates high rates of survival (87). In summary, developing a mechanistic understanding of the regulatory networks for ASC and MBC differentiation, and how these are influenced by selection signals in the GC, is an important area of ongoing research.

## Discussion: Bridging Dynamical Scales in the GC from Molecular Networks to Repertoires

The GC reaction is a regulated synchrony of events, with timescales ranging from minutes for signaling to weeks for the emergence of the repertoire and resolution of the response. It also connects individual genes and signaling proteins within a cell to entire clonal lineages and various cell types inside lymphoid tissues, eventually impacting the whole immune memory repertoire of a person or animal. Further, the GC reaction must be robust to variation and heterogeneity across all these different scales, starting from the distribution of various molecular abundances within different precursor cells, up to populations of mutating pathogens and people with diverse genetics and exposures. Thus, understanding how the dynamics at each of these scales impact each other is critical to learning how high affinity, diverse, and highly specific antibodies from desirable clonal lineages, as well as long term memory, are developed in response to vaccination. In this review, we have addressed some of these multi-scale dynamical phenomena related to B-cells, which are precursors to the effector outputs (ASCs and MBCs) of the GC reaction. There are also similar dynamics associated with Tfh-cells and FDCs or other lymphoid stroma, which are outside the current scope of this piece, but nevertheless of significant interest to vaccinology.

It is evident that besides our vaccine-related successes, we have also acquired extensive immunological knowledge at each scale of the antibody response – about basic B-cell biology, antibody affinity maturation, and functional outcomes such as memory generation within the GC. Many open problems still exist within each scale, such as deciphering which genetic and epigenetic factors in the molecular network interact with each other and how, and the mechanisms by which migrations and interactions in the GC are spatially localized and coordinated. However, the challenge of applying immunology to address the most relevant and immediate challenges for vaccine development begins with the task of bridging these different scales. Here, we briefly outline some of the missing links in connecting the molecular network to cellular interactions and the generation of the repertoire, as targets for future studies.

First and foremost, the coordination between intra-cellular molecular network dynamics and inter-cellular interaction dynamics in the GC has been an area of deep scientific interest for many years, due to the need for precise spatiotemporal control of these processes. An interesting way to view this question is in terms of the relative importance of cell-intrinsic and cell-extrinsic processes. For example, it is known that extrinsic signals drive certain B-cell behaviors, like the case of IgM signaling licensing the light zone phenotype (51), or CD40 signaling driving proliferation (65). Yet, other processes appear to be driven by gene expression dynamics intrinsic to B-cells, such as the cyclic “internal timer” regulating their residence times in the dark zone (31), or the tendency towards ASC or MBC differentiation based on Bach2 abundance (85).

When considering the heritability of molecular network states from founder B-cells, this comparison between cell-intrinsic network dynamics and cell-extrinsic signals can be extended to their impact on the affinity distribution and diversity of the overall antibody repertoire. For example, specific signals and their strengths are implicated in B-cell fate decisions in the GC, such as number of divisions dependent on Tfh signal strengths (63). On the other hand, lineage depths in B-cells have been shown *in vitro* to be determined by the proliferative capacities of their founders (43). This raises the question of whether expansion of a clonal lineage is due to higher affinities, competitiveness, and the resulting signals in each individual B-cell independent of its history, or whether it is simply due to stronger survival and proliferative tendencies inherited across generations from its precursors. Likewise, the likelihood and timing of differentiation into ASCs or MBCs can also be seen as a balance between the affinity-dependent signals to a B-cell and its epigenetically heritable propensity towards a certain fate.

A similar question can also be posed for naïve B-cell founders that seed the GC reaction – whether it is an inherent tendency or a specific signal that drives them to enter the cycling GC B-cell state. It has been shown that the number of founder B-cells that seed the GC response, as well as the resulting clonality over time, is highly variable across GCs (88). Understanding what impacts the number of GC founders, and hence the overall repertoire diversity, is therefore of great interest. Connecting all of this across scales, the repertoire can therefore be considered an emergent outcome of the balance between molecular networks and affinity-dependent interactions, with the nature of this balance yet to be determined in various contexts.

Another way in which molecular networks and signaling can impact the antibody repertoire is through the control of AID expression, which could impact the timing and rate of both SHM and CSR – for example, given that canonical NFκB subunits play a role in inducing AID expression in cycling GC B-cells (77). At later stages, AID levels are downregulated prior to ASC differentiation, due to rising Blimp-1 levels and subsequent cRel repression (89). It is unclear whether the rate of SHM is constant at 1 mutation per 1000bp regardless of the levels of AID, or whether there is a quantitative dependence between AID levels and the number of mutations. The latter hypothesis, where SHM rates are indeed proportional to AID expression, could yield an important lever in shaping the antibody repertoire. This would also be logically favorable from a perspective of the GC reaction as an optimization engine to find the highest affinity antibodies. Increased SHM at the initial stages due to high AID expression would allow rapid exploration of the affinity space. Generating MBCs at this stage would allow quicker searches from a range of starting points in subsequent infections, taking into account the possibility of antigenic drift. Downregulating AID later would then allow fine-tuning of affinity, with small changes around a promising antibody sequence, followed by isotype switching and ASC formation to combat the current antigenic challenge.

Finally, the impact of spatial aspects of GC interactions on the repertoire is often overlooked. While the temporal role of antigen availability has been an important consideration in developing vaccination strategies (8, 10), it may be similarly instructive to consider the impact of spatial distributions of antigen and cells on vaccine outcomes. Encounters between cells in the GC, particularly B-cells and antigen or Tfh-cells, are stochastic outcomes of random walks. Thus, their rate and timing are dependent on volumetric densities within the GC. Intuitively, a high-affinity B-cell needs to collect greater amounts of antigen through the BCR, in order to compete successfully for Tfh-cell help. This means it must search for and collect sufficient quantities of antigen for successful affinity discrimination. However, it would also undergo strong priming for apoptosis in the process, due to pro-apoptotic BCR signaling. The requirement for successful selection then implies that a Tfh-cell encounter should then arrive late enough so that the B-cell has collected enough antigen to receive a selection signal proportional to its affinity, but not so late that the B-cell has irreversibly triggered apoptosis. Hence, selection thresholds become dependent on encounter probabilities for B-cells in the GC, which in turn are functions of the cell densities in the GC and the spatial distribution of antigen. The GC size, as determined by stromal cell expansion and differentiation, now becomes a consideration in determining not only density, but also the carrying capacity of cells or lineages that can be supported at any given time to generate the repertoire. Another topic of interest is whether the network topology of the FDC processes that B-cells migrate along plays a role in organizing their interactions (90), and consequently on the efficiency of affinity maturation. Finally, the statistical distribution of step lengths in B-cell random walks may affect GC outputs. Naïve T-cells are known to switch between Brownian, Levy, or other random walks when encountering antigen-presenting cells in different lymph node regions (91, 92). Similar effects may influence B-cell behaviors within each GC zone.

The difficulty of reproducing the functionality of GCs *in vitro* has been a barrier to studying its cross-scale dynamics. This often necessitates indirect or snapshot studies, which may not provide a complete picture of the transient dynamics that are a feature of the GC. *In vivo* imaging studies have made tremendous inroads into understanding shorter timescales, but may not be able to link phenomena across longer timescales and are limited in undertaking controlled perturbation studies. Genetic perturbations have given us insights into molecular processes in the GC and their impacts on the repertoire, but the results are often very broad. In order to avoid under- or over-estimating these effects, large-scale data at high resolution (such as single-cell level) is needed provide correlative insights, particularly in systems with significant variability, but generating such data remains a challenge.

To provide the missing links between molecular network, cellular interaction, and repertoire scales for better vaccine design, we need to stitch together both experimental and computational methods to probe the dynamics at each scale, including genetic perturbations, live cell and *in vivo* imaging, transcriptome and repertoire sequencing. New experimental tools like organoid cultures (93) can recapitulate GC processes *ex vivo* for longer-term studies in a more accessible setting, also permitting the isolation of specific conditions in cell types or other perturbative studies. Data-driven analyses as alternatives to knockout studies provide correlations that address questions such as where the physiological balance lies between cell-intrinsic and extrinsic aspects. Beyond these, a detailed mechanistic picture is necessary to understand cause and effect within a complex multi-scale system like the GC. This requires multi-scale mathematical modelling that seamlessly integrates known dynamical processes at each scale to explain observed effects at higher scales (64). The power of such models lies in capturing insights from independent studies and connecting them logically to each other, using a uniform and consistent framework. Several efforts have already laid an excellent foundation in this area (30, 94), with room for expansion and improvement as we acquire new data and insights. This systems immunology approach to GC dynamics across scales could bring predictability to GC dynamics, enabling rational design of vaccines for desired outcomes, thus effectively leveraging basic immunological insights for addressing current challenges in vaccinology.

## Author Contributions

This manuscript was conceptualized by HVN and AH. HVN wrote the manuscript and AH edited the manuscript. All authors contributed to the article and approved the submitted version.

## Funding

The studies described in this review were funded by NIH R01AI132731 and R01AI127867 to AH. HVN has been supported by a James S McDonnell Foundation Postdoctoral Fellowship Award in Understanding Dynamic and Multi-scale systems, and a Damon Runyon Quantitative Biology Fellowship from the Damon Runyon Cancer Research Foundation [Award DRQ:11-21].

## Conflict of Interest

The authors declare that the research was conducted in the absence of any commercial or financial relationships that could be construed as a potential conflict of interest.

## Publisher’s Note

All claims expressed in this article are solely those of the authors and do not necessarily represent those of their affiliated organizations, or those of the publisher, the editors and the reviewers. Any product that may be evaluated in this article, or claim that may be made by its manufacturer, is not guaranteed or endorsed by the publisher.
